# Expression of P62 in hepatocellular carcinoma involving hepatitis B virus infection and aflatoxin B1 exposure

**DOI:** 10.1002/cam4.1176

**Published:** 2017-09-21

**Authors:** Xiao Xiang, Hong‐Gui Qin, Xue‐Mei You, Yan‐Yan Wang, Lu‐Nan Qi, Liang Ma, Bang‐De Xiang, Jian‐Hong Zhong, Le‐Qun Li

**Affiliations:** ^1^ Department of Hepatobiliary Surgery Affiliated Tumor Hospital of Guangxi Medical University Guangxi Cancer Institute Hospital Oncology School Guangxi Cancer Center Nanning 530021 China; ^2^ Guangxi Liver Cancer Diagnosis and Treatment Engineering and Technology Research Center Nanning 530021 China

**Keywords:** Aflatoxin B1, hepatitis B virus, hepatocellular carcinoma, NRF2, P62

## Abstract

This study aims to clarify the relationship and mechanism between expression of autophagy‐related protein P62 and prognosis of patients with hepatocellular carcinoma (HCC) involving chronic hepatitis B virus (HBV) infection and aflatoxin B1 (AFB1) exposure. HCC patients who underwent resection were divided into three groups: HBV(+)/AFB1(+) (*n* = 26), HBV(+)/AFB1(−) (*n* = 68), and HBV(−)/AFB1(−) (*n* = 14). The groups were compared in terms of mRNA and protein levels of P62, disease‐free survival (DFS), and overall survival (OS) and the expression of NRF2, Nqo1, and AKR7A3 in P62 high‐expression and low‐expression group. HBV(+)/AFB1(+) group has lower DFS and OS, and higher P62 expression than in the other two groups. P62 expression generally correlated with elevated NRF2 and Nqo1 expression, and reduced AKR7A3 expression. Patients expressing high levels of P62 showed significantly lower DFS and OS rates than patients expressing low levels. HCC involving HBV infection and AFB1 exposure is associated with relatively high risk of tumor recurrence, and this poor prognosis may relate to high P62 expression. High P62 expression activates the NRF2 pathway, promotes tumor recurrence. The downregulation of AKR7A3 also reduced liver detoxification of aflatoxin B1.

## Introduction

Hepatocellular carcinoma (HCC) is a common malignant tumor characterized by insidious onset, diverse etiology, and high mortality 1, 2. HCC risk is particularly high in China, which accounts for approximately 55% of cases worldwide; incidence is particularly high along the southeastern coast 3. Several environmental factors contribute to HCC development 4, 5: more than 70% of HCC patients in Japan and the United States are chronically infected with hepatitis C virus (HCV) 6, while many patients in southern China and sub‐Saharan Africa have consumed aflatoxin B1 (AFB1) in their diet and are chronically infected with hepatitis B virus (HBV) 7. Chronic HBV infection and AFB1 exposure play a key role in the development of HCC in developing countries. It is reported that 5–28% of global HCC cases may be due to AFB1 exposure 8. Vandel studies have shown that chronic HBV infection is associated with AFB1 exposure in some countries with high HCC rates; infection and toxin consumption are two major causes of risk factors for liver cancer and further health problems in incident areas 9, 10 The Guangxi region in southwestern China has high prevalence of HBV 11, 12. Because of the warm and humid climate, many crops, especially maize and peanut, are easily polluted by AFB1. The study shows that people living in the area may have liver risk of injury induced by HBV and AFB1 6.

HBV and AFB1 are thought to promote HCC via similar pathways. AFB1 is the most abundant of the aflatoxins, produced by *Aspergillus flavus* and *Aspergillus parasiticus* 13, 14. AFB1 exposure induces DNA damage and mutagenesis in liver tissue 15, and AFB1 exposure and chronic HBV infection cause liver damage. All these processes increase risk of HCC 8, 16, 17. For example, AFB1‐induced DNA damage can result in an Arg‐to‐Ser mutation at codon 249 in p53 in more than 50% of HCC patients exposed to AFB1 18, 19, 20, which increases risk of HCC 21, 22. HBV, for its part, has been linked to HCC pathogenesis via several molecular pathways involving RB1, methylation of p16INK4a, and amplification of cyclin D1 23. HBV can induce AFB1 to AFB1‐8,9‐epoxide by direct and indirect manner through specific enzyme CP450. HBV originates from chronic hepatitis or the virus itself may promote sensitization of hepatocytes during AFB1 toxicity 9. Metabolites have a damaging effect on DNA, and viral DNA can be more easily integrated into the host genome by acute toxicity of binding proteins. The direct effects of AFB1 and HBV may cause these effects 24. The synergistic effect between AFB1 and HBV is observed by clinical studies of HBV transgenic mice. There is a consistent mechanism to reveal the interaction of HBV infection, which changes the expression of aflatoxin metabolic enzymes 9.

Another factor that may contribute to HCC is P62, which regulates the transcription of genes involved in the cell cycle, cellular proliferation, and apoptosis 25. P62 is also a multifunctional signaling hub and autophagy adaptor that promotes the autophagic degradation of ubiquitinated proteins. P62 accumulates in several liver diseases, including nonalcoholic steatohepatitis and HCC, where it occurs in Mallory‐Denk bodies and intracellular hyaline bodies. Chronic P62 elevation contributes to HCC development by enhancing the proliferation of cancer‐initiating cells and inhibiting their senescence and death.

Here, we analyzed whether P62 expression correlates with prognosis of patients from southern China with HCC involving chronic HBV infection and AFB1 exposure. This may help clarify whether P62 may be involved in HCC pathogenesis in such patients, and whether P62 expression may be a useful prognostic marker. As a secondary aim, we also systematically compared prognosis between patients with or without HBV infection and AFB1 exposure.

## Methods

### Patients and tissue samples

This retrospective study involved a consecutive series of HCC patients who underwent curative liver resection at the Affiliated Tumor Hospital of Guangxi Medical University between February 2013 and February 2014. Curative hepatectomy was defined as complete resection of visible tumors at surgical margins based on histological examination 11, 12, 26.

Fresh HCC tissues were from the Tumor Tissue Bank of the Affiliated Tumor Hospital of Guangxi Medical University. Control tissue samples were taken from patients with histology‐confirmed hepatic hemangioma who underwent liver resection at the Affiliated Tumor Hospital and who were not infected with HBV or HCV.

The study protocol was approved by the Guangxi Medical University Affiliated Cancer Hospital Ethics Committee, and procedures were carried out according to the Declaration of Helsinki (2013 version). Written informed consent was obtained from all patients.

### Patient classification

Tumor stage was determined according to the Barcelona Clinic Liver Cancer (BCLC) staging system. Patients were classified as HBV(+) if their serum contained >1000 copies of HBV DNA and if they were positive for HBsAg, HbeAb (or HbeAg), and anti‐HBc antibody. Patients were defined as HBV(−) if they were negative for HBsAg, HBeAg, and anti‐HBc antibody. Patients were classified as AFB1(+) if they possessed the Arg‐to‐Ser mutation at codon 249 of the p53 gene and if their HCC tissue stained positive for *A. flavus* DNA. Patients negative for both of these markers were defined as AFB1(−). Patients positive for one marker but negative for the other were excluded from the study.

### Follow‐up

All patients underwent periodic follow‐up after liver resection. At 1 month after hepatectomy and every 3 months thereafter, the following tests were performed: serum assay of alpha‐fetoprotein (AFP), chest X‐ray, abdominal computed tomography or magnetic resonance imaging, and abdominal ultrasonography. The last follow‐up was conducted in February 2017.

### Quantitation of P62 mRNA levels

Total RNA was extracted from tissues using Trizol reagent (Invitrogen, Grand Island, NY). Complementary DNA was generated using the PrimeScript^™^ reverse transcription reagent kit (TAKARA, Japan) according to the manufacturer's instructions, then amplified by PCR using the SYBR Premix Ex Taq^™^ Kit (TAKARA) and the P62‐specific primers 5′‐ACCTGAACCCTCTCGTG‐3′ (forward) and 5′‐GTGATGGCTCCCCTTAC‐3′ (reverse). Results were analyzed using the ABI StepOne Plus System (Applied Biosystems, Foster City, CA), and P62 mRNA levels were normalized to those of *β*‐Actin, which was amplified using the primers 5′‐AAGGCCAACCGCGAGAA‐3′ (forward) and 5′‐ATGGGGGAGGGCATACC‐3′ (reverse). All primers were synthesized by Hebei Bohai Biological Processing Development Co., Ltd. (Hebei, China).

### Quantitation of P62 protein levels

Tissues were lysed in RIPA buffer (Solarbio, Beijing, China) containing 1 mmol/l PMSF (Solarbio, Beijing, China), and proteins were collected by centrifugation at 12,000*g* at 4°C for 10 min. Protein concentrations were determined using the BCA protein assay kit (Beyotime, Shanghai, China). Equal amounts of protein (20 *μ*g) were separated by 10% sodium dodecyl sulfate‐polyacrylamide gel electrophoresis and then transferred to polyvinylidene difluoride membranes (Millipore, MA). Membranes were blocked for 2 h with 5% skim milk in phosphate‐buffered saline (PBS) containing Tween‐20 (PBST), and then incubated overnight at 4°C with rabbit monoclonal antibody against P62 (1:1000; Abcam, Cambridge). Actin was also detected as an internal control. After three washes in PBST for 5 min each, membranes were incubated for 2 h at room temperature with goat anti‐rabbit horseradish peroxidase‐conjugated antibody (1:5000; Abcam). Bands were visualized using an Enhanced Chemiluminescence kit (Beyotime, Shanghai, China). All experiments were carried out three times.

### Immunohistochemistry of P62 in tissue sections

Tissues were fixed with 10% formalin, embedded in paraffin, and sections (3 mm) were cut. Sections were deparaffinized in xylene, then rehydrated in a descending ethanol gradient. Antigen retrieval was carried out using pressure cooking in Tris/EDTA (pH 9.0), followed by incubation with 3% hydrogen peroxide for 15 min to block endogenous peroxidase activity. Sections were washed three times with PBS for 3 min each, then incubated at room temperature for 2 h with rabbit monoclonal antibody against P62 (1:800; Abcam). Sections were again washed three times with PBS, incubated at room temperature for 20 min with biotin‐conjugated goat anti‐rabbit secondary antibody (ZSGB, Beijing, China), then stained with 3,3‐diaminobenzidine tetrahydrochloride. P62 protein expression was quantitated based on mean and integrated optical density of antibody staining.

### Statistical analysis

All statistical analyses were performed using SPSS 16.0 (Chicago, IL) and a significance threshold of *P* < 0.05. Continuous data were expressed as mean ± SD and compared between groups using Student's *t* test. Categorical data were analyzed using a two‐sided chi‐squared test or Fisher's exact test, as appropriate. Disease‐free survival (DFS) and overall survival (OS) were analyzed using the Kaplan–Meier method, and the results for different patient groups were compared using the log‐rank test. Univariate analysis and multivariate analysis based on Cox proportional hazards regression was used to identify independent predictors of DFS or OS. To reduce bias, researchers analyzing P62 mRNA and protein levels, P62 immunohistochemistry, recurrence, and survival were blinded to the HBV and AFB1 status of the patients.

## Results

### Study population

**Figure 1:**
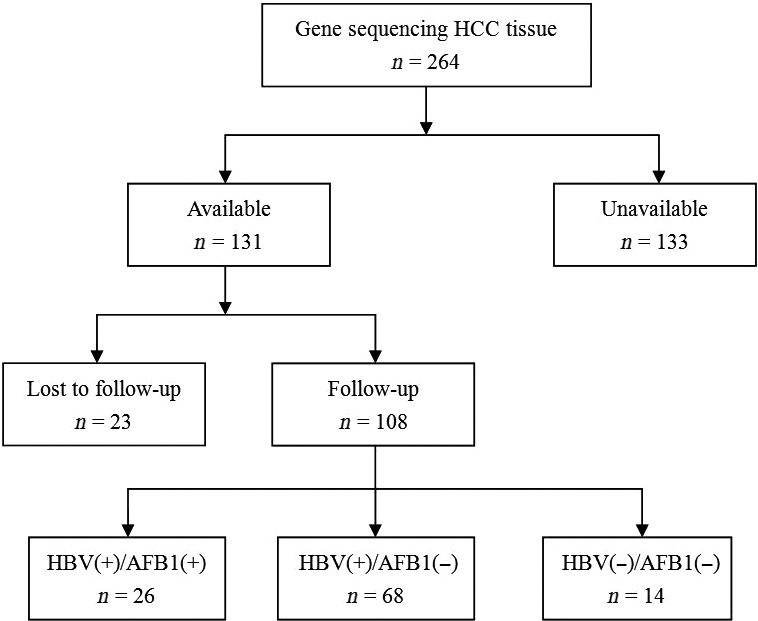
Flowchart of patient selection.

During the study period, 264 HCC patients underwent curative liver resection at our hospital, but 156 (59.0%) had to be excluded because they were infected with HCV (*n* = 7, 2.6%), they had received other anti‐tumor treatments before liver resection (*n* = 60, 22.7%), no fresh tissue samples were available for them (*n* = 56, 21.2%) or they were lost to follow‐up (*n* = 23, 8.7%). The remaining 108 patients were included in the study (Fig. 1). Another 10 samples of normal liver tissue were collected as controls.

### Clinicopathological characteristics

Baseline demographic and clinicopathological data for the 108 patients are shown in Table 1. The patient population consisted of 91 men and 17 women, with a mean age of 49 ± 11 years. Most patients (79.6%) had liver cirrhosis, and all had Child‐Pugh A liver function. BCLC stage was A for 54.6% of patients, B for 27.8%, and C for 17.6%. The patients were subdivided into three groups according to HBV infection and AFB1 exposure status: HBV(+)/AFB1(+) (*n* = 26), HBV(+)/AFB1(−) (*n* = 68), and HBV(−)/AFB1(−) (*n* = 14). Most clinicopathological characteristics were similar among the three groups at baseline.

**Table 1:** Baseline characteristics of patients with hepatocellular carcinoma who underwent liver resection

Variable	Total	HBV(+)/AFB1(+)	HBV(+)/AFB1(−)	HBV(−)/AFB1(−)	*P*
*N*	108	26	68	14	
Gender					0.016
Male	91 (84.2)	24 (92.3)	59 (86.7)	8 (57.1)	
Female	17 (15.8)	2 (7.7)	9 (13.3)	6 (42.9)	
Age, years	49 ± 11	42 ± 10	48 ± 11	54 ± 8	0.146
Tumor size, cm	8 ± 4.5	8.5 ± 5	7.7 ± 4	7.3 ± 3.5	0.623
Tumor number					0.833
Single	71 (65.7)	17 (65.3)	46 (67.6)	8 (57.1)	
Multiple	37 (34.3)	9 (34.7)	22 (32.4)	6 (42.9)	
Cirrhosis					0.767
Absent	86 (79.6)	20 (76.9)	54 (79.4)	12 (85.7)	
Present	22 (20.4)	6 (23.1)	14 (20.6)	2 (14.3)	
HBsAg, +/−	94/14	26/0	68/0	0/14	<0.001
AFP, ng/mL					
≥400	55 (50.9)	16 (61.5)	33 (48.5)	6 (42.9)	
<400	53 (49.1)	10 (38.5)	35 (51.5)	8 (57.1)	
Macrovascular invasion					0.407
Yes	19 (17.6)	4 (15.4)	14 (20.6)	1 (7.1)	
No	89 (82.4)	22 (84.6)	54 (79.4)	13 (92.9)	
BCLC stage					0.041
A	59 (54.6)	15 (57.7)	39 (57.4)	5 (35.7)	
B	30 (27.8)	7 (26.9)	15 (22.0)	8 (57.2)	
C	19 (17.6)	4 (15.4)	14 (20.6)	1 (7.1)	
ALB, g/L	42.3 ± 4.2	41 ± 4.5	42.6 ± 4.6	42 ± 4.7	0.204
PT, sec	13.3 ± 1.3	13.6 ± 1.2	13.2 ± 1.4	12.9 ± 0.8	0.273
ALT, IU/L	45.7 ± 34.5	42.8 ± 28.8	46.1 ± 27.8	34 ± 33.3	0.334
Direct bilirubin, mg/dL	5.9 ± 2.8	5.9 ± 2.3	5.8 ± 2.7	6.1 ± 3.0	0.677
Total bilirubin, mg/dL	12.1 ± 5.3	14.2 ± 5.3	12.1 ± 4.9	10.6 ± 6.4	0.120
Platelet count, *μ*/L	205 ± 91	205 ± 105	203 ± 87	216 ± 95	0.880

Values are *n* or *n* (%) or mean ± SD. ALB, albumin; ALT, alanine aminotransferase; PT, prothrombin time.

### Disease‐free survival

**Figure 2:**
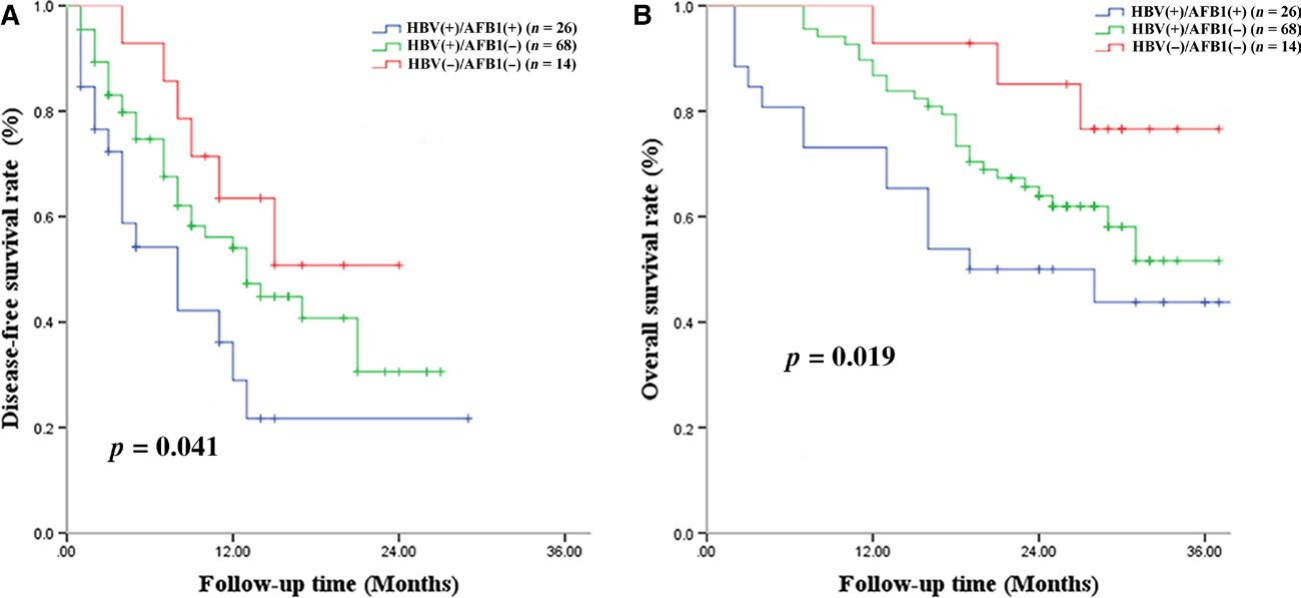
Disease‐free survival and overall survival curves for patients with hepatocellular carcinoma involving chronic hepatitis B virus infection and/or aflatoxin B1 exposure.

A total of 56 (51.8%) patients experienced tumor recurrence within 3 years after liver resection, including 16 (61.5%) patients in the HBV(+)/AFB1(+) group, 34 (50%) in the HBV(+)/AFB1(−) group, and 6 (42.8%) in the HBV(−)/AFB1(−) group. Among all patients, median DFS was 14 months, and DFS rates were 52.4% at 1 year, 38.7% at 2 years, and 30.4% at 3 years. The HBV(+)/AFB1(+) group showed significantly worse DFS rates than the other two groups at 1 year (36.2% vs. 54.1%, 63.5%), 2 years (21.7% vs. 40.7%, 50.8%), and 3 years (21.7% vs. 30.5%, 50.8%) (Fig. 2A). Median DFS was 10 months in the HBV(+)/AFB1(+) group, 14 months in the HBV(+)/AFB1(−) group, and 16 months in the HBV(−)/AFB1(−) group. The HBV(+)/AFB1(−) group showed a significantly lower DFS rate than the HBV(−)/AFB1(−) group at 1 year (54.1% vs. 63.5%), 2 years (40.7% vs. 50.8%), and 3 years (30.5% vs. 50.8%) (Fig. 2A). Median survival time in the two groups was 10 and 14 months. Uni‐ and multivariate analyses identified the following independent predictors of early HCC recurrence: BCLC stage C, ≥2 tumors, and macrovascular invasion **(**Table 3 **)**.

**Table 2:** Baseline characteristics of patients with hepatocellular carcinoma after liver resection (P62 high‐expression versus low‐expression)

Variable	Total	P62 high expression	P62 low expression	*P*
*N*	108	57	51	
Gender				0.054
Male	91 (84.2)	52 (91.2)	39 (76.4)	
Female	17 (15.8)	5 (8.8)	12 (23.6)	
Age, years	49 ± 11	48.9 ± 10.3	49.0 ± 10.9	0.408
Tumor size, cm	8 ± 4.5	8.4 ± 4.6	7.5 ± 3.9	0.542
Tumor number				0.066
Single	71 (65.7)	42 (73.6)	29 (56.8)	
Multiple	37 (34.3)	15 (26.4)	22 (43.2)	
Cirrhosis				0.002
Absent	86 (79.6)	52 (91.2)	34 (66.6)	
Present	22 (20.4)	5 (8.8)	17 (33.4)	
HBsAg, +/−	94/14	55/2	39/12	<0.001
AFP, ng/mL				0.692
≥400	55 (50.9)	28 (49.1)	27 (52.9)	
<400	53 (49.1)	29 (50.9)	24 (47.1)	
Macrovascular invasion				0.989
Yes	19 (17.6)	10 (17.5)	9 (17.6)	
No	89 (82.4)	47 (82.5)	42 (82.4)	
BCLC stage				0.601
A	59 (54.6)	25 (43.8)	34 (69.7)	
B	30 (27.8)	23 (40.4)	7 (13.7)	
C	19 (17.6)	9 (15.8)	10 (19.6)	
ALB, g/dL	42.3 ± 4.2	41.9 ± 4.6	42.3 ± 4.4	0.981
PT, sec	13.3 ± 1.3	13.5 ± 1.3	13.4 ± 1.2	0.342
ALT, IU/L	45.7 ± 34.5	48.7 ± 38.5	43.6 ± 30.7	0.753
Total bilirubin, mg/dL	12.1 ± 5.3	12.12 ± 4.6	12.5 ± 6.0	0.132
Direct bilirubin, mg/dL	5.9 ± 2.8	5.9 ± 2.5	6.0 ± 3.0	0.165
Platelet count, *μ*/L	205 ± 91	205.8 ± 92.8	193.9 ± 84.6	0.515

Values are *n* or *n* (%) or mean ± SD. ALB, albumin; ALT, alanine aminotransferase; PT, prothrombin time.

### Overall survival

During the follow‐up period, 15 patients (57.7%) died in the HBV(+)/AFB1(+) group, 17 (25.0%) in the HBV(+)/AFB1(−) group, and 6 (42.8%) in the HBV(−)/AFB1(−) group. Among all patients, OS rates were 87.7% at 1 year, 68.0% at 2 years, and 48.6% at 3 years; median survival time was 29 months. The HBV(+)/AFB1(+) group showed a significantly lower OS rate than the two other groups at 1 year (71.5% vs. 85.7%, 92.3%), 2 years (50.1% vs. 64.3%, 86.2%), and 3 years (42.5% vs. 49.3%, 77.7%) (Fig. 2B). Median survival time was 20 months in the HBV(+)/AFB1(+) group, 27 months in the HBV(+)/AFB1(−) group, and 31 months in the HBV(−)/AFB1(−) group. The HBV(+)/AFB1(−) group showed a significantly lower OS rate than the HBV(−)/AFB1(−) group at 1 year (85.7% vs. 92.3%), 2 years (64.3% vs. 76.2%), and 3 years (49.3% vs. 67.7%) (Fig. 2B). Median survival time in the two groups was 27 and 31 months. Uni‐ and multivariate analyses identified ≥2 tumors and macrovascular invasion as independent predictors of OS **(**Table 4 **)**.

### P62 expression in HCC tissues

**Figure 3:**
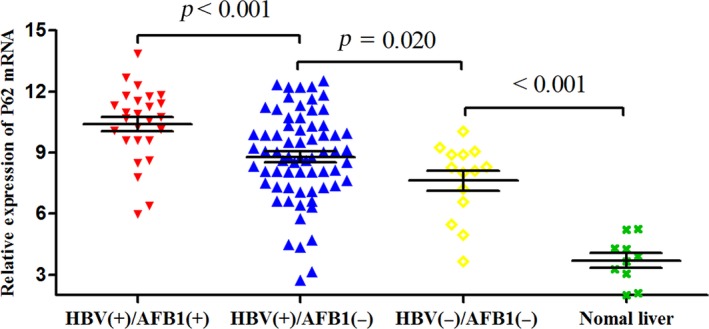
Levels of P62 mRNA based on quantitative reverse transcription‐PCR.

**Figure 4:**
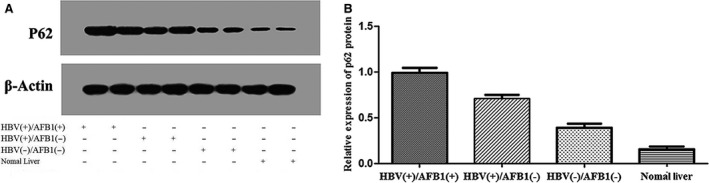
Levels of P62 protein based on western blotting.

We analyzed P62 expression in HCC samples from all 108 patients, as well as 10 samples from normal liver tissue as negative controls. Expression was assessed at the mRNA level using quantitative reverse transcription‐PCR and at the protein level using western blotting and immunohistochemistry. The mean level of P62 mRNA was significantly higher in HBV(+)/AFB1(+) tumor tissues (10.39 ± 1.81) than in HBV(+)/AFB1(−) tissues (8.78 ± 2.19; *P* < 0.001) and HBV(−)/AFB1(−) tissues (7.62 ± 1.83; *P* < 0.001; Fig. 3). In addition, the level in the HBV(+)/AFB1(−) group was significantly higher than the levels in the HBV(−)/AFB1(−) group (*P* = 0.020) and in the normal liver control samples (3.70 ± 1.12, *P* < 0.001; Fig. 3). Similar results were obtained based on measurement of P62 protein levels (Fig. 4A and B): the level was significantly higher in the HBV(+)/AFB1(+) group than in the other two groups, and it was significantly higher in the HBV(+)/AFB1(−) group than in the HBV(−)/AFB1(−) group or normal liver.

**Figure 5:**
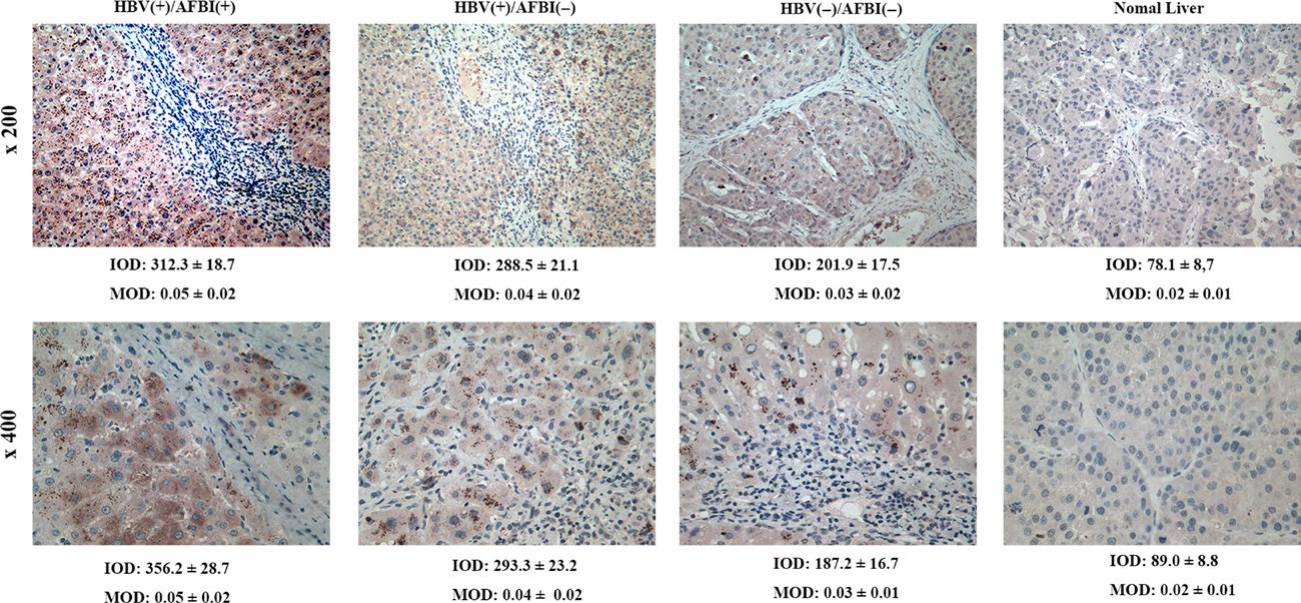
Representative micrographs showing anti‐P62 immunohistochemical staining in cancerous and normal liver tissue. IOD, integrated optical density; MOD, mean optical density (C).

Paraffin‐embedded tissue blocks were available for 90 of the 108 patients, so P62 protein expression was quantitated based on immunohistochemistry. Expression was higher in the HBV(+)/AFB1(+) group than in the other groups, and expression was higher in the HBV(+)/AFB1(−) group than in the HBV(−)/AFB1(−) group or normal liver samples **(**Fig. 5 **)**.

### P62 activates NRF2 signaling pathway

**Figure 6:**
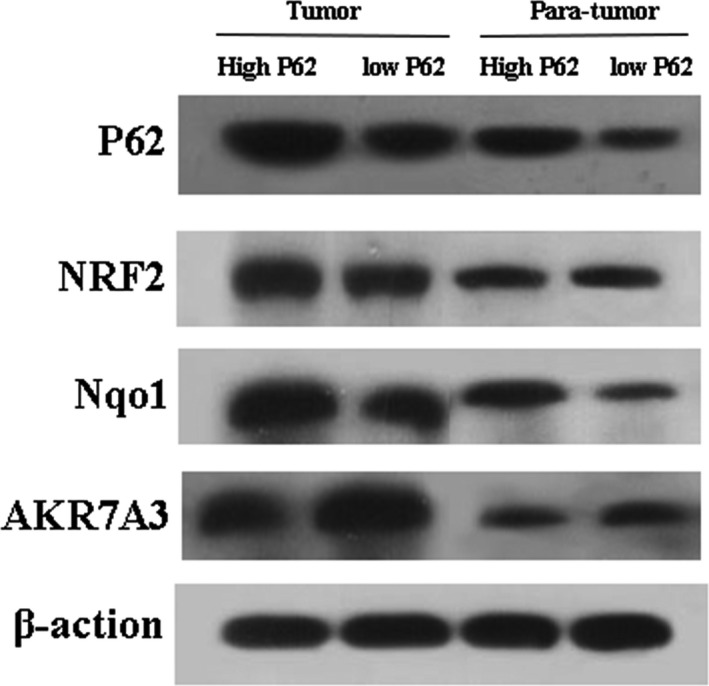
Levels of NRF2, Nqo1, AKR7A3 protein in high or low P62 expression of tumor tissue and para‐tumor tissue.

**Figure 7:**
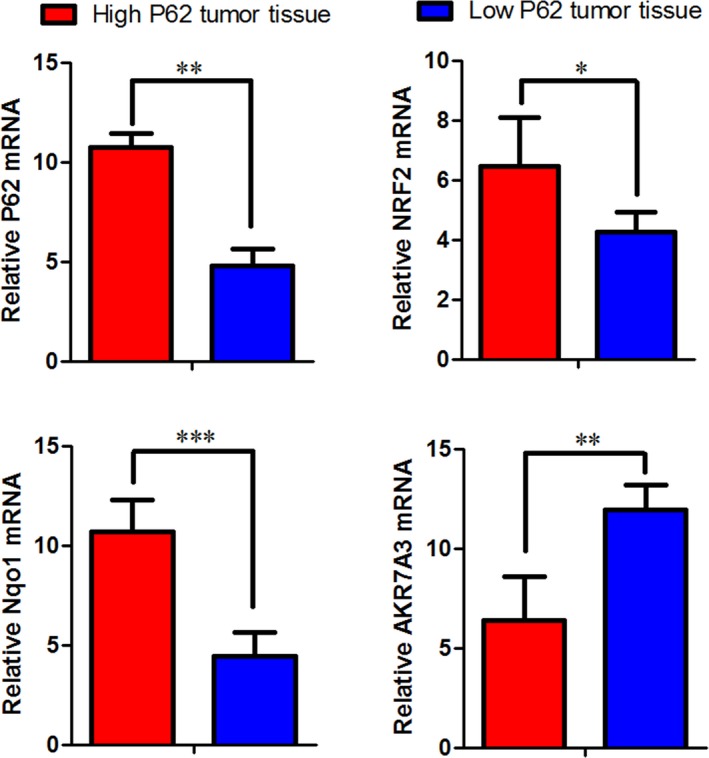
Levels of NRF2, Nqo1, AKR7A3 mRNA in high or low P62 expression of tumor tissue.

Next, we examined the expression of NRF2, Nqo1, AKR7A3 in P62 high‐ expression group and low‐expression group. P62 in high‐expression group increased the expression of NRF2 and Nqo1, reduced expression of AKR7A3. P62 in low‐expression group reduced the expression of NRF2 and Nqo1, increased the expression of AKR7A3 **(**Figs. 6 and 7 **)**.

### P62 expression and DFS

**Figure 8:**
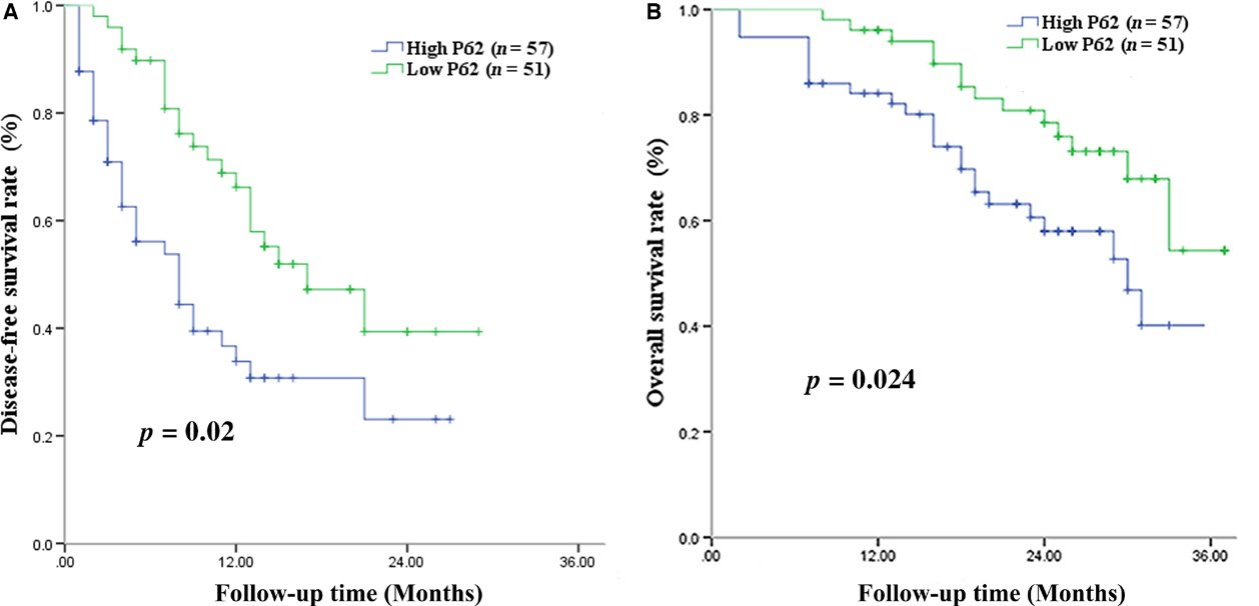
Disease‐free survival and overall survival curves for patients with hepatocellular carcinoma involving chronic hepatitis B virus infection and/or aflatoxin B1 exposure, stratified by high or low P62 expression.

Next, we examined whether P62 expression correlated with prognosis for patients with HCC associated with HBV and AFB1 exposure. Of all 108 patients, 57 (47.8%) were defined as showing high P62 expression and 51 (65.1%) as showing low P62 expression **(**Table 2 **)**. Patients with high‐expressing of P62 showed significantly lower DFS rates than those with low‐expressing at 1 year (33.8% vs. 66.2%), 2 years (23.1% vs. 47.2%), and 3 years (23.1% vs., 47.2%) (Fig. 8A). Median DFS was 7 months among high‐expressing patients and 12 months among low‐expressing patients. Uni‐ and multivariate analyses identified the following independent predictors of HCC recurrence (Table 3): high P62 expression, BCLC stage C, tumor size >10 cm, ≥2 tumors, and macrovascular invasion.

**Table 3:** Uni‐ and multivariate analyses to identify factors influencing tumor recurrence after liver resection in patients with hepatocellular carcinoma

Variable	Univariate			Multivariate		
	Hazard ratio	95% CI	*P*	Hazard Ratio	95% CI	*P*
Age > 60 years	2.551	1.047–6.218	0.039			
Male gender	0.521	0.238–1.142	0.104			
Tumor > 10 cm	2.141	1.087–4.218	0.028	2.311	1.183–4.314	0.031
Multiple tumors	1.859	1.001–3.453	0.050	2.032	1.312–3.642	0.043
AST > 40 U/L	1.370	0.649–2.893	0.409			
Serum AFP > 400 ng/mL	0.904	0.500–1.635	0.739			
ALT > 40 U/L	0.821	0.406–1.662	0.584			
Total bilirubin > 12 mg/dL	0.510	0.129–2.008	0.335			
Direct bilirubin > 7 mg/dL	1.494	0.763–2.924	0.242			
ALB > 40 g/L	0.623	0.337–1.151	0.131			
Platelets > 100 *μ*/L	0.486	0.216–1.095	0.082			
PT >13 sec	1.014	0.536–1.916	0.967			
Macrovascular invasion	2.277	1.226–4.230	0.009	2.373	1.026–4.421	0.011
BCLC stage C	2.304	1.109–4.785	0.025	2.122	1.012–4.594	0.032
High P62 expression	3.682	1.522–5.511	<0.001	3.731	1.586–5.574	<0.001

AFP, alpha‐fetoprotein; ALB, albumin; ALT, alanine aminotransferase; AST, aspartate transaminase; BCLC, Barcelona Clinic Liver Cancer; PT, prothrombin time; 95% CI, 95% confidence interval.

### P62 expression and OS

High‐expressing patients showed significantly lower OS rates than low‐expressing patients at 1 year (84.1% vs. 90.7%), 2 years (59.7% vs. 80.9%) and 3 years (38.5% vs. 56.1%) (Fig. 8B). Median OS was 26 months in the high‐expressing group and 31 months in the low‐expressing group. Uni‐ and multivariate analyses identified the following independent predictors of OS: high P62 expression, tumor size >10 cm, ≥2 tumors, and macrovascular invasion **(**Table 4).

**Table 4:** Uni‐ and multivariate analyses to identify factors influencing overall survival after liver resection in patients with hepatocellular carcinoma

Variable	Univariate			Multivariate		
	Hazard ratio	95% CI	*P*	Hazard Ratio	95% CI	*P*
Age > 60 years	1.412	0.549–3.634	0.475			
Male gender	1.029	0.434–2.440	0.947			
Tumor size > 10 cm	1.395	0.614–3.168	0.427			
Multiple tumors	1.468	1.254–2.524	0.046	1.631	1.185–2.803	0.028
AST >40 U/L	1.887	0.768–4.638	0.166			
Serum AFP > 400 ng/mL	1.207	0.613–2.375	0.586			
ALT > 40 U/L	0.441	0.180–1.081	0.073			
Total bilirubin >20 mg/dL	0.239	0.052–1.105	0.067			
Direct bilirubin >7 mg/dL	1.503	0.687–3.292	0.308			
ALB >40 g/L	0.831	0.426–1.620	0.586			
Platelets > 100 *μ*/L	1.307	0.550–3.105	0.544			
PT > 13 sec	1.024	0.483–2.174	0.950			
Macrovascular invasion	1.664	2.305–4.042	0.047	1.618	2.264–4.033	0.041
BCLC stage C	1.499	0.711–3.160	0.288			
High P62 expression	1.491	1.254–3.947	0.024	1.447	1.242–3.965	0.021

AFP, alpha‐fetoprotein; ALB, albumin; ALT, alanine aminotransferase; AST, aspartate transaminase; BCLC, Barcelona Clinic Liver Cancer; PT, prothrombin time; 95% CI, 95% confidence interval.

## Discussion

HBV infection and AFB1 exposure may contribute to more than 80% of HCC cases in the Guangxi area of southern China 6. We found that among HCC patients, chronic HBV infection was associated with significantly shorter DFS and OS, which were even shorter among patients with chronic HBV infection as well as prior AFB1 exposure. We also found that among HCC patients, P62 expression was significantly higher in the presence of chronic HBV infection and even higher in the presence of HBV infection as well as prior AFB1 exposure. These findings highlight the prognostic impact of the well‐known HCC risk factors of HBV and AFB1, and they provide strong evidence that P62 expression may be a novel prognostic indicator for HCC patients who have undergone hepatic resection.

Our findings suggest that chronic HBV infection and AFB1 exposure can affect prognosis of HCC patients, and that the two risk factors can synergize. Both HBV and AFB1 act primarily through pathways involved in detoxification, drug metabolism, antigen processing, glycolysis, and anti‐apoptosis 27. HBV infection causes oxidative stress and chronic inflammation in liver cells, leading to accumulation of reactive oxygen species (ROS) 8. HBV infection may also downregulate detoxification‐related proteins 28, making HBV‐infected liver cells more susceptible to carcinogens such as AFB1. The increased risk of HCC from AFB1 exposure in HVB‐infected patients can increase cell turnover allowing replication of AFB1‐DNA adducted cells. Increased cell turnover can result in selection of cell populations with increased expression of oncogenes‐like p62 or mutated tumor suppressor genes such as TP53 29. Second, AFB1 metabolism results in two damaging species epoxides which can form DNA adducts and aldehydes that can lead to excess reactive oxygen species (ROS) leading to indirect damage of DNA and other cellular biomolecules 30. AKR7A3 is important for AFB1 detoxication and downregulation of AKR7A3 could be contributory to HCC but it should be noted that epoxide dehydrogenases and GSTs (glutathione S‐transferases) are also central to AFB1 metabolite detoxication 31.

We found high P62 expression to be associated with significantly higher risk of recurrence and death in HCC patients following hepatectomy. P62 is a major component of intracellular hyaline bodies, Mallory‐Denk bodies, and hybrid inclusions 32, which are hallmarks of chronic liver diseases that substantially increase risk of HCC 33. In fact, studies suggest that P62 accumulation is required for progression from premalignancy to malignancy, most likely because it prevents HCC‐initiating cells from dying under oxidative stress. As a result, such cells can accumulate multiple oncogenic mutations 34, 35. These studies point to a role for P62 in HCC initiation and progression, which is consistent with our results on the prognostic impact of P62 expression. HCC is not the only neoplasia associated with P62 accumulation: the protein has also been implicated in renal cell carcinoma 36, 37, endometrial cancer 38, and benign adenomas in autophagy‐deficient livers 39.

In this study, we focused on the Arg‐to‐Ser mutation at codon 249 in the p53 coding sequence because this mutation has been found in more than 50% of HCC patients living in regions where AFB1 exposure is high 20, 21, 40. Nevertheless, the profile of p53 mutations associated with AFB1 exposure can vary across HCC populations, reflecting differences not only in AFB1 exposure but also in the specific mutations triggered by AFB1 metabolism 41, 42, 43, 44. A study of 397 HCC patients from the same Guangxi area as this study, for example, found that while codon 249 was a mutational hotspot, mutations in exons 4, 5, 6, 7, 8, and 9 were detected 45. Thus, while our results should be considered reliable for this particular subtype of AFB1‐exposed HCC patient, they may not be generalizable to other patient subtypes. Future work should look at patients with a broader range of AFB1‐induced mutations.

HBV infection can induce autophagosome formation by inhibiting the fusion between autophagosomes and lysosomes to promote autophagy. HBx is to maintain infection and replication essential virus protein 46. HBx can induce autophagic liver cell lines by upregulating the expression of Beclinl 47. HBx can trigger autophagy by direct interaction with type III phosphatidylinositol 3‐kinase (PtdIns3K), which is beneficial to viral replication. These pathways may activate the P62 protein located downstream of the autophagic pathway 48. In addition, p62 is a stress‐inducible protein that may be induced by some inflammatory factors that are released by AFB1 infection, and its expression is regulated by Nrf2 34. p62 overproduction is sufficient to phosphorylate p62 Keap1, resulting in a positive feedback loop activation of accelerated Nrf2. Thus, autologous maturation in HCC patients with persistent inflammation of HBV and AFB1 will result in phosphorylation of p62 and subsequent activation of Nrf2 49.

NRF2 is regulated by translation after E3 ubiquitin ligase Keap1, which induces NRF2 ubiquitination and proteasome degradation. After activation of activated oxygen or electrophiles, Keap1 is oxidized and cannot bind to the newly translated NRF2, which stabilizes and enters the nucleus to activate the gene containing the antioxidant response element 49. NRF2 is the primary activator of P62 transcriptional regulation and posttranscriptional regulation in malignant liver tissue is activated during chronic liver inflammation and oxidative stress. In turn, p62 can activate NRF2, constituting two self‐amplifying autoregulatory loops 50. Ohta et al.'s study 51 showed that KEAP1 inactivation mutations occur in lung cancer and HCC, and activation of genes that inhibit NRF2 inhibition of Keap1 in lung, Skin, and liver cancer 52. Schulze et al.'s study found that NRF2 and KEAP1 mutations occurred in 14% of HCC specimens and were thought to be driving mutations 53. There is growing evidence that the Keap1‐Nrf2 pathway is one of the major cellular defense mechanisms of antioxidant and electrophilic stress 34, 49. When cytotoxic ubiquitination components such as mitochondrial damage and invasive microbes occur, phosphorylated p62 binds to Keap1 with high affinity and NRf2 ubiquitin ligase complexes 54. This binding inhibits Keap1‐driven ubiquitination of Nrf2, which in turn leads to the stabilization of Nrf2, which is then transferred to the nucleus and induces the transcription of many cytoprotective genes encoding antibody proteins, detoxification enzymes, and multidrug transporters. The ubiquitinated structure and phosphorylated p62 and Keap1 complexes are autophagically degraded, leading to the elimination of cytotoxic components, thereby protecting the tumor cells 55.

AKR7A3, which locates on chromosome 1p36, belongs to the AKRs superfamily,whose function is responsible for the detoxification of aflatoxin B1—a potent hepatocarcinogen 29. These enzymes were also reported to play important roles in nuclear receptor signaling, cellular metabolism, inflammatory responses, osmoregulation, endobiotic and xenobiotic detoxification, and hormone synthesis 32. Chow et al. reported that AKR7A3 is frequently downregulated in HCC, associating with poor overall survival rate, elevated AFP, and poor differentiation of HCC 56. Jin et al. reported that AKR1 and AKR7 are involved in the development of cancers such as breast, lung, liver, colorectal, and prostate cancers 33. Hlava et al. reported that in breast cancer, high expression level of AKR7A3 significantly associates with longer disease‐free survival 34.

We found that macrovascular invasion and multinodular tumors were associated with poorer prognosis after hepatectomy. This is consistent with other studies 57, 58. Macrovascular invasion increases risk of HCC recurrence, which is the primary cause of death among HCC patients after hepatic resection 59, 60, 61. Multinodularity has been associated with earlier tumor recurrence and worse prognosis 62, 63, 64. We did not find serum AFP level or diabetes mellitus to be independent predictors of survival, which contrasts with previous studies 65, 66, 67, 68.

The analyses in this study were possible because of our ability to recruit HCC patients who could be rigorously verified as having HBV infection and/or AFB1 exposure. Despite this advantage over other studies in the literature, our work does have some important limitations. First, all the patients came from a single center, and our center is a referral hospital, so most patients have moderate or advanced liver cancer. These factors may increase the risk of selection bias. In addition, our sample was fairly small and we did not have a HBV(−)/AFB1(+) group for comparison. Larger, preferably multisite studies on P62 expression and prognosis are needed.

In conclusion, our results suggest that chronic HBV infection and AFB1 exposure significantly worsen prognosis of HCC patients after hepatic resection, and our results implicate P62 in this worsened prognosis. It may be that HCC patients with at least one of the three risk factors of chronic HBV infection, AFB1 exposure, or high P62 expression would benefit from more extensive resection, postoperative active adjuvant therapy, and more frequent follow‐up.

## Conflicts of Interest

The authors have declared that no competing interests exist.
